# An Experimental Investigation of Sewage Sludge Gasification in a Fluidized Bed Reactor

**DOI:** 10.1155/2013/479403

**Published:** 2013-12-25

**Authors:** L. F. Calvo, A. I. García, M. Otero

**Affiliations:** ^1^IMARENABIO, University of León, Avenida de Portugal 41, 24071 León, Spain; ^2^CESAM, Department of Chemistry, University of Aveiro, Campus de Santiago, 3810-193 Aveiro, Portugal; ^3^Department of Applied Chemistry and Physics, University of León, Campus de Vegazana, 24071 León, Spain

## Abstract

The gasification of sewage sludge was carried out in a simple atmospheric fluidized bed gasifier. Flow and fuel feed rate were adjusted for experimentally obtaining an air mass : fuel mass ratio (A/F) of 0.2 < A/F < 0.4. Fuel characterization, mass and power balances, produced gas composition, gas phase alkali and ammonia, tar concentration, agglomeration tendencies, and gas efficiencies were assessed. Although accumulation of material inside the reactor was a main problem, this was avoided by removing and adding bed media along gasification. This allowed improving the process heat transfer and, therefore, gasification efficiency. The heating value of the produced gas was 8.4 MJ/Nm, attaining a hot gas efficiency of 70% and a cold gas efficiency of 57%.

## 1. Introduction

Sewage sludge originates from the process of treatment of municipal wastewater. In parallel with the enhancement and increase of sewage treatment plants to comply with more and more exigent environmental policies, production of sewage sludge has also increased and it is expected to increase even more. Although sludge treatment and disposal should be considered as an integral part of wastewater treatment, its handling is still one of the most significant challenges in wastewater management [1].

During the last years, former options for sewage sludge disposal, including ocean dumping, landfill, or disposal on agricultural land, have been forbidden, restricted, or became less acceptable from an environmental point of view. Consequently, cost-effective and environmentally friendly alternatives to these disposal means are needed. There is not an only and best management option for sewage sludge but it must be planned according to each sludge properties and local circumstances. Thus, under certain conditions, thermal processes may be appropriate options since they can be used for the conversion of large quantities of sewage sludge into useful energy [2]. Among thermal processes, gasification, that is, the thermal conversion of sewage sludge to combustible gas and ashes under a net reducing atmosphere, provides an attractive alternative to the most extended incineration [3]. Gasification not only accounts with all the advantages of incineration regarding sewage sludge management, such as complete sterilization, large volume reduction, or odour minimization, but also circumvents its main problems. The need for supplemental fuel, emissions of sulfur oxides, nitrogen oxides, heavy metals, and fly ash, and the potential production of dioxins and furans, which may be produced during incineration as a consequence of the oxidizing atmosphere [4], are avoided by sewage sludge gasification. A main advantage of sewage sludge gasification is that a high-quality flammable gas may be obtained, so it can be directly used for electricity generation or for supporting the drying of sewage sludge [5] and also may be employed as raw material in chemical synthesis processes [6]. Indeed, as for biomass gasification, a main difficulty about sewage sludge gasification is the presence of tar and dust in the synthesis gas produced, which may cause problems in process equipment and/or in turbines and engines for gas distribution [7–11]. In any case, gasification is considered a waste-to-clean energy technology [12].

Theory on sewage sludge gasification and a description of the process were reported by Dogru et al. [13] and also by Fytili and Zabaniotou [1] in their review on thermal processing of sewage sludge. Since Garcia-Bacaicoa et al. [14] first published a work on sewage sludge gasification, encouraging results have been reported, mainly during the last decade [3, 5, 6, 9–11, 15–18]. However, as recently highlighted by de Andrés et al. [6], comparatively with the large number of references that can be found in scientific literature regarding biomass gasification, experimental works on sewage sludge are relatively scarce. Thus, the overall objective of this work was to identify characteristics of sewage sludge gasification in atmospheric fluidized-bed gasifier. To this end, analyses of the mass transformation efficiency, gas composition, cold gas efficiency, and tar production were carried out.

## 2. Materials and Methods

### 2.1. Fuel Characterization

Sewage sludge obtained from the Oakland (California) sewage treatment plant was dried, granulated, and stored in an airtight container until use. Sieve distribution of homogenized dried granulated sewage sludge is shown in Table 1. As can be observed, the most frequent size is 40 < mesh < 20, which accounts for 38.12%, particles larger than 40 mesh representing 22.4% of the sample.

Before gasification, sewage sludge was analyzed for the following properties.

Moisture content was determined gravimetrically by the oven-drying method (ASTM D 3173, ASTM E 871). Triplicate samples, typically weighing 20 to 80 g each, were obtained from the container and air-dried at 104 ± 3°C in an atmospheric oven to constant weight, normally obtained within 24 hours.

Higher heating value at constant volume (HHV) was measured using an adiabatic oxygen bomb calorimeter (via the equivalent methods ASTM D 2015, ASTM E 711, or ASTM D 5468). Triplicate samples of approximately 1 g each were split from batches prior to each test and analyzed using the Parr Model 1241 calorimeter with Model 1720 controller. Fuel was sampled in 1 g amounts, pelletized in a hand press to 12.7 mm diameter, and oven-dried to constant weight at 104 ± 3°C prior to analysis.

Proximate determinations were made according to modified procedures from ASTM D 3172 through D 3175 (Standard Practice for Proximate Analysis of Coal and Coke); E 870 (Standard Methods for Analysis of Wood Fuels), D 1102 (ash in wood), and E 872 (volatile matter in wood); and the methods for refuse derived fuel (RDF)—E 830 (ash) and E 897 (volatile matter).

Triplicate samples, approximately 1 g each, split from the main sample batch were dried at 104 ± 3°C and analyzed. Ash concentration was determined at 575°C for 2 hours in an atmospheric pressure air muffle. This temperature is that specified by ASTM for RDF and is slightly below the minimum temperature specified for wood (580°C).

Volatile concentration was determined under anaerobic conditions using a modified method for sparkling fuels in which samples in covered nichrome crucibles were placed in the front part of the open muffle furnace preheated to 950°C for 6 minutes to dispel volatiles over a period of more gradual heating and then brought to completion in the closed furnace during an additional 6 minutes, removed, and cooled under desiccant while still covered and weighed immediately.

Percent fixed carbon (dry basis) was computed by subtracting percent ash (dry basis) plus percent volatile matter (dry basis) from 100.

All crucibles were prefired at the test temperature (575 or 950°C) before use in order to remove any moisture or volatiles prior to each determination.

Ultimate analysis was carried out following standard methods.

### 2.2. Gasification Tests

#### 2.2.1. Gasification Reactor

**Figure 1:**
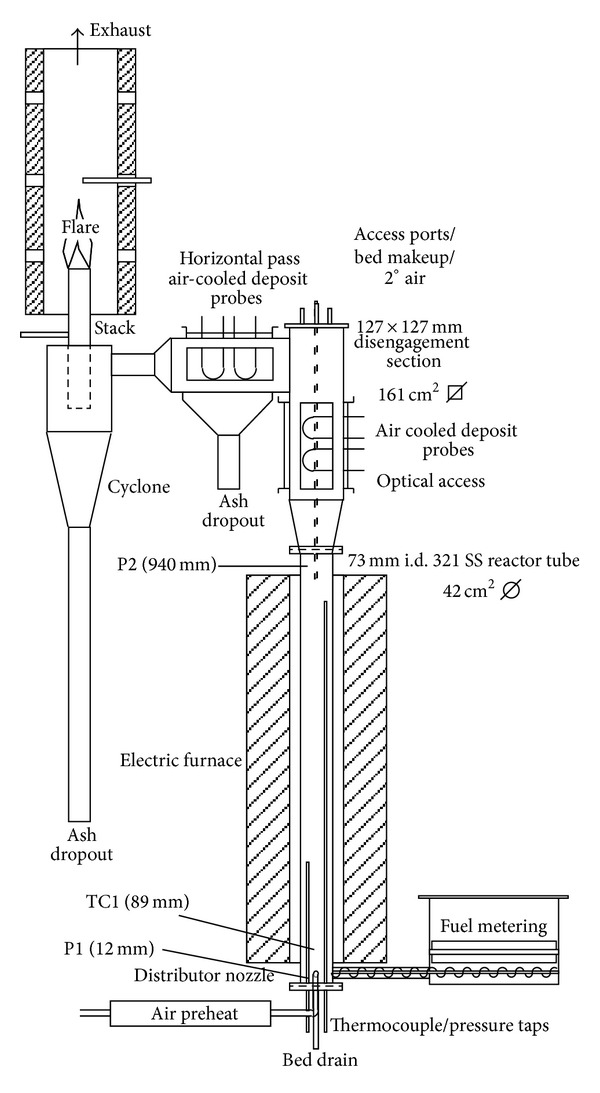
Fluidized-bed reactor (TC = thermocouple, P = pressure tap, and D = disengagement thermocouple).

The reactor used for sludge gasification, which is schematically shown in Figure 1, is an atmospheric pressure rig, with a main reactor column of 73 mm inside diameter and 1 m in length. The main column discharges into a 127 mm^2^ disengagement zone for internal recirculation of particles. Sewage sludge was fed to the reactor at a controlled rate using a custom-designed belt feeder driven by variable speed stepper motor. Fuel was injected in bed using a high-speed stainless steel auger. Fuel metering was controlled by the speed of the belt, while the auger was used solely for fuel injection. The fuel feeder and hopper were pressurized using a small amount of purge air (called secondary air) to prevent back-flow of reaction products into the fuel feeder.

The reactor column is made of 321 stainless steel and is surrounded by an electric furnace used to preheat the reactor. The electric furnace is automatically controlled using reactor wall temperature.

Fluidizing, or primary, air is preheated through a series of parallel electric heaters before being discharged through the distributor nozzles in the bottom of the bed. The bottom of the reactor terminates in a blind flange through which bed discharge, thermocouple, and pressure taps are inserted. The reactor was constructed in such a way that it could be rapidly disassembled for inspection and cleaning.

Above the furnace, the reactor expands into the disengagement section with four times the cross-sectional area of the main bed. Larger fuel and bed media particles are disengaged from the gas flow at this point and returned to the bed along the wall of the reactor column. Situated at the top of the disengagement section is a removable lid through which temperature, pressure, and bed make-up taps are inserted.

After the disengagement section the flow turns 90°. An ash drop-out is located at this position. A cyclone is situated past the horizontal pass and discharges separated particles through the bottom. Gas is flared at the cyclone exit stack under natural aspiration inside a refractory lined exhaust duct ported to receive sample inlets. Gas and fly-ash samples are drawn from the cyclone exit.

Thermocouples, pressure transducers, outputs from continuous gas analyzers, and other electronic transducers are automatically recorded using a multichannel data logger communicating with a personal computer. A ten-second sampling interval is used.

#### 2.2.2. Bed Material

Bed material selected for the experiments was alumina-silicate sand (NARCO Investocast 60 grain). Fresh, screened bed material was used for each test. The required amount of fresh bed media was introduced in the gasifier before the gasification experiments. The initial mass of the bed was weighed and recorded. In any case, fresh media can be added through a top access port during operation and, also, bed material may be removed along gasification. After each test, spent bed was removed by dropping the lower flange plate and bed was captured. Any residue in the bed was determined from loss on ignition in an air muffle furnace at 575°C.

#### 2.2.3. Gas Analysis

Continuous gas sampling/analysis was accomplished by a Leeds & Northup gas analyzer (analyzes CO, CO_2_, and H_2_) and by a Panametrics O_2_ analyzer for identifying the possible existence of leaks in the sample.

Grab samples were collected in glass for permanent gases, primarily CH_4_ via GC. Primarily sampling locations for gases were taken at the cyclone exit.

#### 2.2.4. Alkali Vapors Sampling

**Figure 2:**
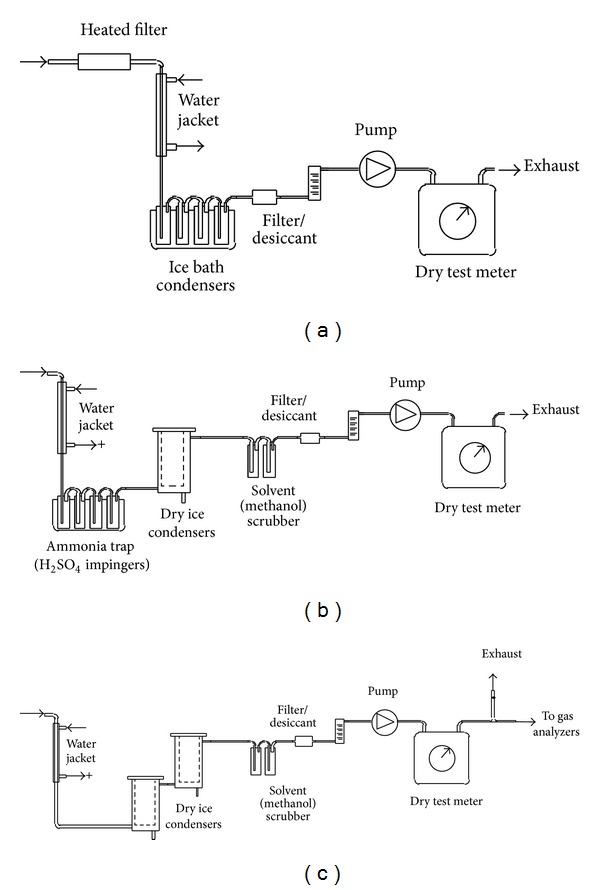
(a) Alkali sampling train; (b) ammonia sampling train; (c) tar sampling train.

The alkali sampling train is schematically shown in Figure 2(a). The sample was extracted through a heated 5 *μ*m sintered stainless steel filter to separate particles. The filter was maintained at the same temperature as the gas at the sampling point so as to reduce errors in the determination of the alkali partitioning between the gas and particle phases. Filtered gas passed through a water-jacketed condenser and ice-bath cooled impinger train, a desiccant pump, a rotameter or mass flow meter, and a dry-test meter. This liquid volume was recorded and a sample was analyzed for the species of interest.

#### 2.2.5. Ammonia Sampling

Nitrogenous species other than NO_*x*_ (principally ammonia) were also measured via an absorption train. Ammonia sampling was conducted according to the methods of Ishimura [19], Furman et al. [20], and Blair et al. [21]. Similar to the alkali sampling, sample stream was drawn through a water-jacketed condenser and then through a set of ice-bath impingers filled with sulfuric acid 0.1 M solution. After the test, liquid volume from the impingers was weighed and recorded. An ion selective electrode (sensitive to ammonium) was used to measure (NH_4_^+^) and hence ammonia. This train is schematically shown in Figure 2(b).

#### 2.2.6. Tar

Tar was condensed in a set of condensers using dry-ice in ethanol to provide the low temperatures required. Methanol scrubbers were used after the condensers to capture some of the lighter hydrocarbons. The gas volume and tar weight were measured.  Figure 2(c) schematically shows this train.

#### 2.2.7. Generalized Test Procedures

Sewage sludge was injected into the preheated bed at a rate controlled by the speed of the belt on the fuel feeder. Reactor heating was controlled automatically by the electric furnace around the reactor. Temperatures and pressures were monitored throughout each test. Total air and fuel flow rates were monitored, as were total flows through each of the sampling trains. Grab samples of gas were taken at frequent intervals for gas chromatography analysis. After gas sampling was finished, the fuel and air supplies were cut off from the reactor, and the reactor was cooled.

Posttest sampling was carried out after cooling of the reactor. The entire bed was recovered through the bottom reactor flange and analyzed for ashes and volatiles. Ash/char collected from the horizontal pass and cyclone was collected and put at the same test. Full mass and energy balances were completed as a check on analysis quality and to provide information about material and energy conversion efficiencies. Deposit probes were removed and depositions of the line samples were analyzed for the intended species after recording their weight. Ammonia and alkali were analyzed using an ionspecific electrode. Tar production was determined gravimetrically.

## 3. Results and Discussion

### 3.1. Fuel Properties

Properties of the sludge used in this work are shown in Table 1. Properties of sewage sludge depend on sludge treatment and on sludge origin. Anyway, compared with sludge tested by other authors [6, 9–11, 15, 18], it can be observed that values in Table 1 are in the same order. Although ashes content is lower [6, 9–11] or similar [22] to that of some sewage sludge already used for gasification experimentation, it is high compared with that of biomass normally used for gasification. Volatiles content is in the range of the values found in the literature for sewage sludge to be used as fuel for gasification [10, 13, 22] while different biomass-based fuels used for gasification in the literature may have higher [23–25] or lower [26] volatiles content.

### 3.2. Gasification Tests

Since gasification is a partial combustion, the process requires less oxygen than a complete combustion. Considering the sludge chemistry analysis shown in Table 1, a sludge elementary molecule (C : H : O) could be written as CH_1.48_O_0.31_ and, then, complete combustion would be represented by the following equation:

(1) CH1.48O0.31+1.215(O2+3.76N2) →CO2+0.74H2O+4.568N2ΔHf  0=−422.03 kJ/mol

The air mass : fuel mass ratio (A/F) is 6.08 for complete combustion. For gasification, that ratio should be, in theory, between 0.2 and 0.4 [27]. Air flow and fuel feed rate were the parameters changed for experimentally obtaining a 0.2 < A/F < 0.4. Two tests were carried out, test I and test II, Table 2 showing the corresponding main gasification parameters. A complete analysis for these tests was done, including gas analysis.

During test I, the accumulation of material inside the reactor was a main problem. As it may be seen in Table 2, the difference between bed media weight after and before test I was 1589.74 g. In order to avoid material accumulation and to improve the process heat transfer and, therefore, gasification efficiency, bed media was removed and fresh bed added along test II. As a result, there were no any accumulation problems during test II.

Ash and volatile analysis was carried out for depositions from the horizontal pass and cyclone and for the removed bed.  Table 3 shows the obtained results. The ash content of those depositions was similar for tests I and II, the percentages being 85.05 and 80.91%, respectively. These values indicate that there were fuel particles in the removed bed, although less in test II than in test I because of having removed bed material during test II. Volatiles content is similar in the samples taken from cyclone and removed bed for both experiments. However, in the samples from horizontal pass, the percentage of volatiles in test I is 1.6 higher than in test II. This is due to the high content of not completely burned fuel particles inside the reactor in test I.

The mass transformation efficiency was determined by a mass balance and results are shown in Table 4. Percentage of raw produced gas is higher in test II than in test I, with values of 79.69 and 40.30%, respectively. That means that removed bed material during the test is convenient for this fuel if retention of particles inside the reactor want to be avoided. Depositions from pass horizontal are higher in test I for the same reason.

Produced gas was analyzed in tests I and II and Table 5 shows the average composition obtained.

The high value of nitrogen concentration was due to gasification using air. This means that, although the process is cheaper than with pure oxygen, the quality of the produced gas is lower. The sum of the H_2_, CO, and CH_4_ percentages in tests I and II is 44 and 40.7%, respectively; these values are higher than values found in literature [16]. Seeing that the high heating values of CO, H_2_, and CH_4_ are 12.87, 12.99, and 41.22 MJ/Nm^3^, respectively, it can be deduced that produced gas from test I was better, in terms of energetic quality, than that produced in test II, which can be owing to the fact that flow air was higher in test II.

For determining the energy efficiency of the system, an energy balance was calculated, results being shown in Table 4 together with the corresponding energetic efficiency parameters.

The heating value of the produced gas was 9.33 and 8.42 MJ/m^3^, in tests I and II, respectively, values which are quite close. However, it must be taken into account that, although heating value of the produced gas is slightly higher in test I, the mass gas obtained in this test is almost twice lower, with respect to inputs, than in test II. Common values for cold gas efficiency are between 0.45 and 0.67 [28]. This range was attained in test II, but not in test I, which may be related to the accumulation of particles inside the reactor and derived problems.

For test I, 87.6% of the gas energy was obtained due to its chemical energy and the most important contribution was from methane (42.52%). Although hydrogen is the component that presents the highest enthalpy (141.90 MJ/kg), its contribution in chemical energy was only of 34.07% because, as may be seen in Table 5, its volume percentage was 21%. For test II, chemical energy is equal to 81.4% of total energy. In this case the most important contribution was also from methane, with 38.16%. Finally, the lost energies in test I were higher (61% with respect to inputs) due to the same reasons explained before.

### 3.3. Tar Train

Dust, water, and tars constitute depositions of tar train. Dust percentage in produced gas is known by the depositions in the heater filter placed in alkali train. The difference in weight before and after the train is the dust accumulated on it.

The tar quantities condensed in the tar train during tests I and II are shown in Table 5. The mass of tar from test I was 1.4 times higher than that from test II. The total tar values, with respect to the sewage sludge fed, are comparatively lower than those obtained by [22] during the gasification of sewage sludge in a spouted bed reactor. In any case, a gas cleaning system for tar removal would be needed for industrial applications. Between wet and hot gas cleaning, the latter should be preferred since it really destroys the tars instead of transferring them to a liquid phase, which would need further and expensive treatment [29]. Also, for energy efficiency reasons of the whole process, the hot gas cleaning is a promising method [30] and several authors have recently studied this sort of method with successful results [30–33].

## 4. Conclusions

Gasification of sewage sludge was carried out using air, which is cheaper than pure oxygen. A high quality gas (H_2_, CO, and CH_4_ summed up to 40.7–44%) with a heating value of 8.42–9.33 MJ/Nm^3^, low in tar content (0.6 g/m^3^) and cold gas efficiency of 57% was obtained. Comparatively with published results on the gasification of sewage sludge [34], these values are outstanding and demonstrate that gasification of sewage sludge, which may be carried out in a real straightforward way, is an option for the valorization of sewage sludge.

## Figures and Tables

**Table 1:** Sieve distribution and properties of sewage sludge.

	% Total
Particle size (mesh)	
>200	3.57
200 < 100	2.30
100 < 40	16.52
40 < 20	38.12
20 < 14	22.04
<14	17.64
Elemental analysis	
C (%)	36.2
H (%)	4.5
N (%)	5.6
S (%)	1.1
Cl (%)	0.1
O (%)	14.7
Proximate analysis	
Moisture (%)	7.9
Ash (%)	37.9
Volatiles (%)	55.1
Fixed carbon (%)	7.1
Heating value	
HHV^1^ (MJ/kg)	15.4

^1^HHV: high heating value.

Except moisture, all values are on dry basis.

**Table 2:** Gasification parameters in tests I and II.

	Test I	Test II
Wet fuel burned (g)	4521.7	11743.9
Wet fuel feed rate (g/s)	8.94	12.52
Reactor preheating (°C)	850	850
Primary air preheating (°C)	300	300
Primary air (L/min)	15	20
Bed material	Al-Si	Al-Si
Fresh bed material (g)	866	433
Removed bed material (g)	2455.74	433 + 128.6 + 1782.6^1^
Ash horizontal pass (g)	1261.6	193.1
Ash cyclone (g)	115.3	113.2
Alkali sampling train		
Bottle 1 (g)	46.6	97.1
Bottle 2 (g)	3.8	50.7
Bottle 3 (g)	0.9	−121.5
Bottle 4 (g)	0.5	0.5
Desiccant (g)	1.9	2.8
Filter paper (g)	0.0947	0.052
Filter cake (g)	3.3	0.4
Gas analyzed (m^3^)	0.0972	0.1151
Time in alkali train (min)	26	123
Average flow rate through tar train (L min^−1^)	3.738	0.936
Ammonia sampling train		
Bottle 1 (g)	109.9	85.6
Bottle 2 (g)	11.5	8.3
Bottle 3 (g)	2.3	2.1
Bottle 4 (g)	0.7	0.1
Desiccant (g)	2.9	6
Filter paper (g)	0.0066	0.0003
Gas analyzed (ft^3^)	4.631	14.101
Time in ammonia sampling train (min)	26	123
Average flow rate through ammonia sampling train (L min^−1^)	5.044	3.246
Tar sampling train		
Condenser 1 (g)	180.3	147.3
Bottle and tubing (g)	94.6	92.4
Line 1 (g)	2	3.5
Condenser 2 (g)	75.2	113.6
Line 2 (g)	0.7	1.4
Impinger 1 (g)	−6.8	−219
Impinger 2 (g)	7.8	3.2
Line 3 (g)	0	0.1
Desiccator (g)	2.6	12.5
Filter paper (g)	0.0197	0.0025
Gas analyzed (m^3^)	0.3955	0.7547
Time in tar train (min)	44	123
Average flow rate through tar train (L min^−1^)	8.989	6.136

^1^Added bed during the test.

**Table 3:** Ash and volatile analysis of depositions from horizontal pass, from cyclone, and from removed bed for tests I and II.

	Ash from horizontal pass deposition (%)	Ash from cyclone depositions (%)	Ash from removed bed (%)	Volatiles from horizontal pass deposition (%)	Volatiles from cyclone depositions (%)	Volatiles from removed bed (%)
Test I	80.84	79.66	85.05	9.10	13.80	4.08
Test II	82.43	79.15	80.91	5.64	12.49	4.94

**Table 4:** Mass balance, energy balance, and energetic efficiency parameters for tests I and II.

	Test I		Test II	
	Mass (g)	Input %	Mass (g)	Input %
*Mass balance *				
Inputs				
*m*_*a*_	1032.54	16.08	3618.27	22.91
*m*_df_	4131.97	64.36	10733.92	67.96
*m*_*m*_	389.73	6.07	1009.98	6.39
*m*_*b*_	866	13.49	433	2.74
Outputs				
*m*_hp_	126.16	19.65	238.06	1.51
*m*_*c*_	115.3	1.80	114.57	0.73
*m*_rc_	2455.79	38.25	2854	18.07
*m*_*g*_	2587.55	40.3	12588.54	79.69

	Test I		Test II	
	Energy (MJ)	Power (W)	Energy (MJ)	Power (W)

*Energy balance *				
Inputs				
Fuel	64.0282	24253	166.2960	22533.3
Air	0.1745	66.1	0.6429	87.1
Heaters	1.2804	485	1.6421	222.5
Outputs				
Lost	39.2756	14877.1	51.8074	7020.0
Gas	26.3075	9927.1	116.581	15822.9

	Test I		Test II	

*Energetic efficiency *				
Hot gas efficiency (HGE)	0.41		0.70	
Cold gas efficiency (CGE)	0.34		0.57	
% lost with respect to inputs	0.60		0.31	
% lost with respect to fuel	0.61		0.31	

*m*_*a*_: air mass; *m*_df_ dry fuel mass; *m*_*m*_: fuel moisture mass; *m*_*b*_: fresh bed mass; *m*_hp_: ash from horizontal pass mass; *m*_*c*_: ash from cyclone mass; *m*_rc_: removed bed mass; *m*_*g*_: raw produced gas mass.

**Table 5:** Average concentration (%v/v) of O_2_, CO, CO_2_, H_2_, CH_4_ and N_2_ in produced gas from tests I and II and tar produced in each test.

	Test I	Test II
Produced gas		
O_2_	0.6	0.4
CO	14.6	13.4
CO_2_	11.4	11.1
H_2_	21.0	20.7
CH_4_	8.4	6.7
N_2_	34.1	36.0
Total	**90.1**	**88.2**
Produced tar		
Total depositions (g)	283.75	289.07
Dust (g)	12.21	2.62
% moisture	95.58	98.94
Total tar (g)	0.3348	0.4413
Tars (g/m^3^)	0.846	0.585
